# Inhibitory Effect of ATP on Cytochrome c Oxidase Depends on Electron Entry Pathways by TCA Cycle Metabolites

**DOI:** 10.3390/cells15090811

**Published:** 2026-04-29

**Authors:** Madeline Günther, Valeria Pakic, Petra Weber, Anke Veit, Carsten Culmsee, Ardawan J. Rastan, Annegret P. Busch, Sebastian Vogt

**Affiliations:** 1 Cardiovascular Research Laboratory, University of Marburg, Baldingerstraße, 35043 Marburg, Germany; madeline.guenther@staff.uni-marburg.de (M.G.); pakic@students.uni-marburg.de (V.P.); petra.weber@staff.uni-marburg.de (P.W.); anke.veit1@web.de (A.V.); rastan@uni-marburg.de (A.J.R.); 2 Department of Cardiac Surgery, University Hospital of Giessen and Marburg, 35043 Marburg, Germany; 3 Center for Applied Pharmacy, University Hospital of Giessen and Marburg, 35043 Marburg, Germany; annegret.busch@uk-gm.de; 4 Institute of Pharmacology and Clinical Pharmacy, University of Marburg, 35043 Marburg, Germany; carsten.culmsee@staff.uni-marburg.de

**Keywords:** cytochrome c oxidase, ATP-dependent inhibition, respiratory control, TCA cycle metabolites, complex I and II, mitochondrial ROS, cardiac mitochondria

## Abstract

The ATP-dependent inhibition of cytochrome c oxidase (CytOx, complex IV of the electron transport chain) is the second mechanism of respiratory control adjusting mitochondrial respiration in order to prevent excessive electron flow and reactive oxygen species (ROS) production. Here, we investigate how tricarboxylic acid (TCA) cycle metabolites and the subsequent complex I or complex II activities influence this regulatory mechanism. Therefore, CytOx activity was assessed by the oxygen consumption rate after cytochrome c (Cyt c) titration to stimulate complex IV activity in isolated rat heart mitochondria (RHM) and permeabilized AC16 cells. Mitochondrial membrane potential (Δψm) and ROS formation were analysed by flow cytometry. Our results show that TCA cycle intermediates differed in their impact on CytOx activity and subsequent ROS formation. NADH-linked substrates such as α-ketoglutarate, glutamate and malate increased respiratory capacity, but preserved ATP-dependent control of CytOx, indicating that elevated electron supply alone does not necessarily abolish ATP sensitivity. In contrast, succinate, which feeds electrons directly into complex II, strongly increased respiration causing the loss of ATP-dependent respiratory control in both model systems. Despite this strong respiratory effect, succinate induced only modest changes in mitochondrial membrane potential in isolated mitochondria, whereas permeabilized cardiomyocytes exhibited reduced polarization accompanied by increased superoxide formation. Together, these findings demonstrate that the effectiveness of ATP-dependent CytOx inhibition is influenced by TCA cycle activity and depends on the site of electron entry into the respiratory chain. Thus, substrate-dependent modulation of respiratory control links metabolite availability to mitochondrial redox regulation in cardiac cells.

## 1. Introduction

Mitochondrial respiration must adapt continuously to meet cellular energy demands while maintaining redox balance and preventing the formation of excessive ROS [1]. Respiratory control is classically explained by Mitchell’s chemiosmotic theory, whereby oxygen consumption is regulated by the availability of ADP [2]. When ADP levels are high, ATP synthase activity increases, promoting electron transport and oxygen consumption, while dissipating the proton gradient and lowering the mitochondrial membrane potential. Conversely, when ADP levels are low, proton flux through ATP synthase into the mitochondrial matrix decreases, leading to mitochondrial membrane hyperpolarisation and reduced electron transport. This coupling between ADP availability, mitochondrial membrane potential, and respiratory flux is the classical mechanism of respiratory control.

In addition to this chemiosmotic regulation, mitochondrial respiration can also be controlled directly at the level of CytOx [3]. In this context, ATP binds allosterically to CytOx, promoting phosphorylation-dependent structural changes that inhibit enzyme activity and reduce oxygen consumption [4,5,6]. Unlike the classical ADP-dependent mechanism, this ATP-mediated inhibition operates at the enzymatic level and is considered independent of mitochondrial membrane potential [3]. Accordingly, it has been proposed as a feedback mechanism that slows respiration when cellular energy demand is low, thereby limiting excessive electron flux and suppressing ROS formation [7,8,9]. Evidence of this regulatory function has been obtained in isolated enzymes [4], isolated mitochondria [9], and intact cardiac tissue [10]. Together, these findings indicate that CytOx exerts dual regulatory control over mitochondrial respiration by sensing the intramitochondrial ATP/ADP ratio and modulating electron flux [11].

However, the effectiveness of this inhibitory state is highly dependent on the metabolic and signaling context. Calcium-dependent dephosphorylation of CytOx can switch off the ATP-mediated inhibition, resulting in an increased membrane potential and enhanced ROS production [3,7]. Additionally, changes in electron supply, such as increased cytochrome c availability [12] or nitric oxide signaling [13,14], can increase enzyme turnover and shift respiratory control towards upstream processes.

Despite increasing insights into respiratory control at the level of CytOx, mitochondrial respiration also depends on the balance between electron supply and electron transfer. The generation of reducing equivalents, entering the respiratory chain via CI or CII, is determined by substrate availability and TCA cycle flux. Consequently, changes in TCA cycle metabolism and electron supply subsequently, may alter the extent to which downstream regulatory mechanisms, including allosteric inhibition, determine respiratory activity. It is unclear whether ATP-dependent inhibition of CytOx depends on specific substrate conditions, or relies on the mode of electron entry into the respiratory chain.

In line with this, restriction of electron flux at the level of complex IV itself is not necessarily neutral for upstream electron transfer. Under certain conditions, such a shift creates a state of increased electron pressure in the respiratory chain that influences ROS formation and, in principle, permit reverse electron transport at complex I [15,16,17]. It remains unclear whether ATP-dependent inhibition of CytOx serves to stabilize mitochondrial function or, depending on metabolic context, promotes upstream redox imbalance.

This question is particularly relevant for tissues with high and rapidly changing energy demands such as the heart. In such tissues, mitochondrial respiration must adapt continuously to shifts in substrate availability, which determine the supply of reducing equivalents to the respiratory chain. In this context, TCA-related metabolites such as α-ketoglutarate, glutamate, malate or succinate, enter the respiratory chain at distinct sites rather than modulating electron delivery to CytOx via factors such as the availability of cytochrome c. Therefore, differences between substrates that supply electrons primarily via NADH-linked pathways and those that enter respiration downstream of complex I, may influence not only respiratory capacity, but also the functional impact of ATP-mediated control at complex IV.

In the present study, we therefore investigated how selected TCA-related metabolites, modulate ATP-dependent inhibition of CytOx. Using isolated rat heart mitochondria and permeabilized human AC16 cardiomyocytes, we analysed CytOx activity, mitochondrial membrane potential, and ROS formation under defined ATP/ADP conditions in the presence of these substrates. We aimed to determine whether substrate-dependent electron supply modulates the effectiveness of ATP-dependent respiratory control and thereby influences mitochondrial redox balance.

## 2. Materials and Methods

### 2.1. Animals

Male Wistar rats (7–8 weeks of age upon arrival) were obtained from Charles River Laboratories, Erkrath, Germany. Rats (200–400 g) were housed in polypropylene cages with free access to a standard diet and tap water at all times. All animals were maintained at 24 ± 2 °C and 55 ± 20% humidity on a reverse phase 16 h light/8 h dark cycle. All work detailed in this manuscript was performed under the § 8a Animal Protection Act, reference V54191020/15cMR20/16. Rats were euthanized by decapitation using a guillotine (Ex-2/2023), and the hearts were immediately excised and placed in ice-cold isolation buffer (250 mM sucrose, 20 mM HEPES, 1 mM EGTA, and 0.2% fatty acid-free bovine serum albumin adjusted to pH 7.4 with KOH at 4 °C). Additional washing steps with isolation buffer were performed to remove blood until the solution was clear.

### 2.2. Isolation of Intact Rat Heart Mitochondria

After excision, hearts were chopped into small pieces in 10–15 mL of ice-cold isolation buffer and homogenized in 30–40 mL of the same buffer using a Teflon potter (Kobe KG, Marburg, Germany). The homogenate was first centrifuged at 9000× *g* for 10 min, and the resulting supernatant transferred into a fresh falcon tube, followed by a centrifugation step at 13,000× *g* for 15 min to pellet the mitochondria. The pellet was then washed by resuspension in 5 mL of ice-cold isolation buffer and centrifuged again at 13,000× *g* for 10 min. The final mitochondrial pellet was resuspended in ~200 μL of isolation buffer and stored on ice for further use within 3–4 h. Protein concentration of the mitochondrial preparations was determined using the BCA assay with bovine serum albumin as a standard. Subsequently, mitochondrial suspensions were adjusted to a working concentration of 100 mg/mL, and 0.5 µL of this suspension (corresponding to 50 µg mitochondrial protein) was applied per well in all Seahorse measurements.

### 2.3. Cell Culture

Human AC16 cardiomyocytes (Cytion, Heidelberg, Germany) were cultured in DMEM/Ham’s F12 (Capricorn, Ebsdorfergrund, Germany), supplemented with 15 mM HEPES, 12.5% fetal bovine serum (S0615, Merck, Darmstadt, Germany), 1% penicillin/streptomycin and 2.5 mM L-glutamine.

The cells were maintained at 37 °C in a humidified incubator with 5% CO_2_ and passaged every 2–3 days by trypsinisation up to passage 10.

### 2.4. Compound Preparation

Salts of TCA metabolites were dissolved in MAS buffer (70 mM sucrose, 210 mM mannitol, 10 mM KH_2_PO_4_, 5 mM MgCl_2_, 2 mM HEPES, 1 mM EGTA) with stock concentrations of 100–200 mM. Afterwards, pH was adjusted to 7.2 for all solutions using KOH at room temperature. For experiments, substrates were applied at a final concentration of 5 mM unless otherwise indicated.

### 2.5. CytOx Kinetics

Isolated rat heart mitochondria (0.5 µL per well) were suspended in mitochondrial assay solution (MAS; 70 mM sucrose, 210 mM mannitol, 10 mM KH_2_PO_4_, 5 mM MgCl_2_, 2 mM HEPES, 1 mM EGTA; pH 7.2 adjusted with KOH at 25 °C) supplemented with 0.2% BSA, 0.5 mM malate, 18 mM ascorbate, 10 mM phosphoenolpyruvate (PEP), and 10 U/mL pyruvate kinase. Subsequently, 180 µL of the mitochondrial suspension were dispensed into each well of the XF plate and centrifuged at 4000× *g* for 10 min to attach mitochondria to the wells. During this centrifugation step, the Seahorse cartridge was loaded with the reduced cytochrome c solutions at the desired concentrations. Reduced cytochrome c stock solutions were freshly prepared for each injection port by dissolving cytochrome c (bovine heart, C2037, Sigma-Aldrich, Darmstadt, Germany) in MAS buffer (70 mM sucrose, 210 mM mannitol, 10 mM KH_2_PO_4_, 5 mM MgCl_2_, 2 mM HEPES, 1 mM EGTA, pH 7.2 with KOH at 25 °C) supplemented with 2 mM ascorbate to maintain it in the reduced state. Stock solutions were kept on ice until further use. Then, the respective TCA cycle substrates, and inhibitors (as indicated in each experiment), ADP (1 mM final) and ATP (10 mM final) were applied to the mitochondria in the XF wells immediately before starting the assay. Real-time measurement of CytOx activity was monitored by changes in the oxygen consumption rate (OCR) of isolated mitochondria after sequential injection of cytochrome c (Cytc) at 25 °C. Quantification of CytOx activity was performed from the respective flux analysis based on the magnitude of OCR increase following each cytochrome c injection.

For analysis of CytOx activity in AC16 human cardiomyocytes, cells were seeded at a density of 5000 per well in Seahorse XF cell culture microplates. AC16 cells were then allowed to adhere and grow for 48 h under standard culture conditions.

Prior to the assay, the culture medium was removed and the cells were washed once with MAS buffer. The cells were then permeabilised with 100 µg/mL saponin (SAE0073, Sigma-Aldrich, Merck, Darmstadt, Germany) in MAS buffer for three minutes at room temperature. Following permeabilisation, the permeabilisation medium was removed and cells were maintained in MAS buffer supplemented with the respective TCA cycle metabolites, as indicated for each experiment. Buffer composition, metabolite and Cyt c concentrations were identical to those used for isolated mitochondrial measurements.

### 2.6. Mitochondrial Membrane Potential

Mitochondrial membrane potential (ΔΨm) was assessed using the fluorescence indicator TMRE (Thermo Fisher Scientific). Therefore, mitochondria were resuspended in PBS at a volume of 100 µL. Then, TMRE was added to the cell suspension at a final concentration of 100 nM and samples were analysed immediately. Flow cytometry was conducted using a Thermo Fisher Attune NxT flow cytometer (Fisher Scientific GmbH, Schwerte, Germany) equipped with a 488 nm excitation laser and a 690/50 nm bandpass filter for emission detection. A total of 10,000 events were recorded per sample. The proportion of TMRE-positive mitochondria was quantified by the Attune™ NxT Software (Version 7.1, Thermo Fisher Scientific, Waltham, MA, USA); results are presented as fold-change relative to untreated control.

### 2.7. Mitochondrial ROS Production

Mitochondrial reactive oxygen species production was assessed using MitoSOX Red (Molecular Probes, Thermo Fisher Scientific). Isolated mitochondria (0.5 µL) were resuspended in 100 µL PBS/sample. MitoSOX Red was added to the cell suspension at a final concentration of 1.25 μM and mitochondria were assessed immediately at Ex/EM 488/690 using a Guava Luminex flow cytometer (Luminex Munich GmbH, Munich, Germany) or a Thermo Fisher Attune NxT flow cytometer (Fisher Scientific GmbH, Schwerte, Germany). A total of 5000 events were recorded per sample. Flow cytometry data were analyzed using GuavaSoft 3.3 software (Luminex, Munich GmbH) or the Attune™ NxT Software (Thermo Fisher Scientific, Waltham, MA, USA). Mitochondrial ROS production was quantified as the percentage of MitoSOX-positive events within the gated mitochondrial population.

### 2.8. FACS Analysis of Mitochondrial Membrane Potential and ROS in Permeabilised AC16 Cells

For FACS-analysis, cells were harvested by trypsinisation and washed once with mitochondrial assay solution (MAS) buffer containing 70 mM sucrose, 210 mM mannitol, 10 mM KH_2_PO_4_, 5 mM MgCl_2_, 2 mM HEPES and 1 mM EGTA (pH 7.2, adjusted with KOH). The cells were then resuspended in MAS buffer at a density of 1 × 10^6^ cells per 5 mL, and kept in suspension until measurement.

For each measurement, 100 µL of the cell suspension was transferred to a 1.5 mL FACS tube. The cells were permeabilised by the addition of saponin (final concentration 20 µg/mL) and incubated for 5 min at room temperature. The indicated substrates were then added to a final concentration of 5 mM, either in the absence or presence of ADP (1 mM) or ATP (10 mM).

To analyse the mitochondrial membrane potential (ΔΨm), TMRE with a final concentration of 100 nM was added. The cells were analysed using an Attune NxT flow cytometer (Thermo Fisher Scientific), which was equipped with a 488 nm excitation laser and a 690/50 nm emission filter.

Mitochondrial superoxide formation was assessed using MitoSOX Red at a final concentration of 1.25 µM. The cells were processed as described above, except that TMRE was replaced with MitoSOX. Fluorescence was detected using 488 nm excitation and an emission filter set to 690 nm.

For each condition, 5000 cells per sample were recorded. Data acquisition and analysis were performed using Attune NxT software (Thermo Fisher Scientific). Cells were gated by their percentage of TMRE- or MitoSOX-positive cells relative to the gated cell population.

### 2.9. Statistical Analysis

All data are presented as the mean ± standard deviation (SD). Statistical comparisons between treatment groups were performed using two-way ANOVA, followed by Bonferroni’s test for the three-condition comparisons (Ctrl, ADP, ATP) and Sidak’s test when additional substrate conditions were analysed. A *p*-value of less than 0.05 was considered statistically significant. All calculations were performed and visualized using GraphPad Prism software (Version 8.3.0, GraphPad Software, La Jolla, CA, USA).

## 3. Results

### 3.1. ATP-Dependent Inhibition of CytOx Suppresses Respiration, Restores Membrane Potential, and Reduces ROS Formation

**Figure 1:**
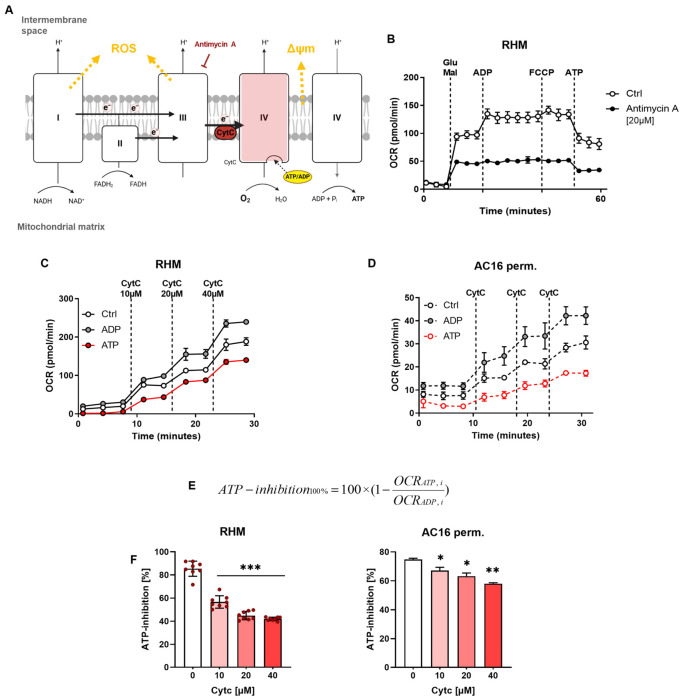
ATP-dependent inhibition of CytOx is the second mechanism of respiratory control. (**A**) Electron flow through the mitochondrial respiratory chain, showing electron entry via complex I (CI) or complex II (CII) into the ubiquinone pool and subsequent transfer through complex III (CIII) to cytochrome c (Cyt c) and complex IV (CIV). CIV reduces O_2_ to H_2_O and contributes to the proton gradient across the inner mitochondrial membrane. ATP binding to CIV mediates allosteric inhibition. (**B**) Seahorse extracellular flux analysis of isolated rat heart mitochondria (RHM) showing decreased glutamate/malate/ADP-supported respiration (5 mM/0.5 mM/1 mM) following ATP (10 mM) injection. (**C**) Rat heart mitochondria were incubated in assay buffer supplemented with 0.5 mM malate, 18 mM ascorbate, 10 mM phosphoenolpyruvate (PEP) and 10 U/mL pyruvate-kinase at room temperature for 15 min. ADP (1 mM) and ATP (10 mM) were applied immediately before the measurement and real-time measurement of CytOx activity was monitored by changes in the OCR of RHM after sequential injection of Cyt c. (**D**) OCR in permeabilized AC16 cardiomyocytes during stepwise Cyt c titration under Ctrl, ADP (1 mM), or ATP (10 mM) conditions. Cells were permeabilized prior to substrate and nucleotide addition. (**E**,**F**) ATP-dependent inhibition was determined for the indicated Cyt c concentration for both RHM and permeabilized AC16 cells. Data are shown as the mean ± SD of four replicate samples per condition. *** *p* < 0.001, ** *p* < 0.01, * *p* < 0.05 compared to untreated control, ANOVA Bonferroni’s test.

To investigate ATP-dependent regulation of CytOx, oxygen consumption rate (OCR) was measured in isolated rat heart mitochondria under defined substrate conditions (Figure 1A,B). In the presence of glutamate/malate and ADP, mitochondria displayed robust respiration. Subsequent addition of ATP markedly reduced OCR, consistent with ATP-mediated inhibition of CytOx. Inhibition of electron transfer to complex IV by antimycin A suppressed overall oxygen consumption, confirming that the observed ATP effect occurs within the respiratory chain.

To further characterize this regulatory mechanism, exogenous Cyt c was titrated to increase electron supply to CytOx (Figure 1C). Under ADP conditions, OCR increased continuously with rising Cyt c concentrations. In contrast, ATP significantly suppressed Cyt c-stimulated respiration. Quantification revealed that ATP-dependent inhibition was strongest at low Cyt c concentrations (~80–90%) and declined at higher Cyt c levels (~40–50% at 40 µM), indicating that enhanced electron delivery partially counteracts ATP-mediated respiratory control (Figure 1E,F).

Comparable results were obtained in permeabilized AC16 cardiomyocytes (Figure 1D,F), demonstrating that ATP-dependent regulation of CytOx is preserved in a cellular context.

**Figure 2:**
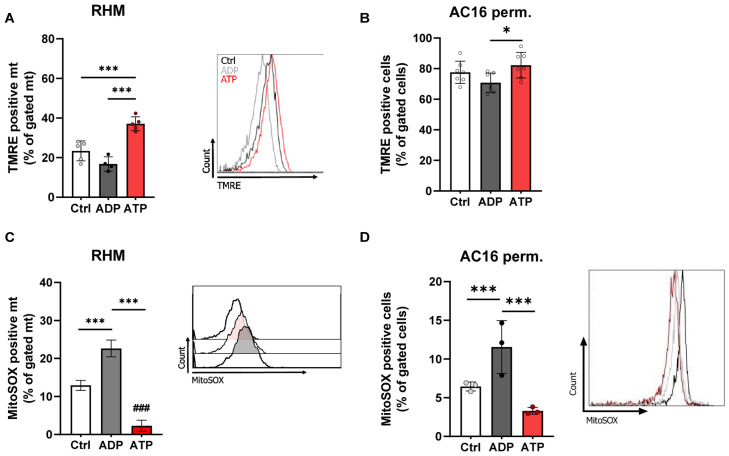
ATP-dependent inhibition of CytOx reduces mitochondrial ROS formation while preserving membrane potential. (**C**,**D**) Mitochondrial membrane potential (ΔΨm) and ROS and were measured by TMRE and MitoSOX fluorescence in presence of ADP (1 mM) or ATP (10 mM) within 5 min in RHM (**A**,**C**) and permeabilized cells (**B**,**D**). All experiments were independently repeated at least three times and are presented as the mean ± SD. ### *p* < 0.000, *** *p* < 0.001, * *p* < 0.05 compared to untreated control, ANOVA Bonferroni’s test.

To assess functional consequences of ATP-dependent inhibition, mitochondrial membrane potential (ΔΨm) and superoxide formation were analyzed (Figure 2). ATP significantly increased TMRE fluorescence relative to ADP in RHM and permeabilized AC16 cells, indicating membrane hyperpolarisation (Figure 2A,B). In parallel, mitochondrial ROS production was reduced under ATP-supplemented conditions (10 mM ATP) in both model systems (Figure 2C,D). These findings show that ATP-dependent inhibition of CytOx not only limits respiratory flux, but also preserves mitochondrial redox state.

### 3.2. System-Dependent Modulation of ATP-Dependent Respiratory Control by CI-Linked Substrates

**Figure 3:**
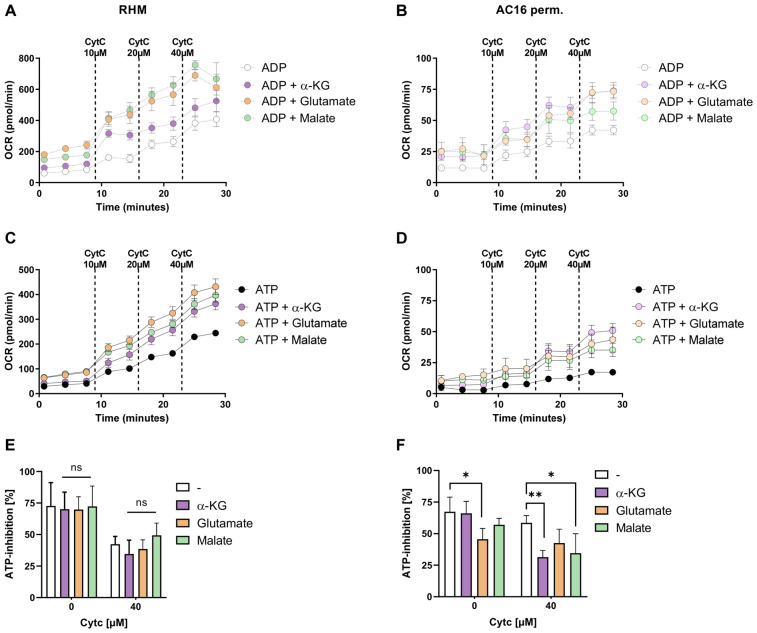
CI-driven electron supply differentially modulates ATP-dependent CIV inhibition in isolated mitochondria and AC16 cells. (**A**,**B**) Oxygen consumption rate in RHM (**A**) and permeabilized AC16 cardiomyocytes (**B**) during stepwise titration of Cyt c (10–40 µM) under ATP- or ADP-supported conditions (10 mM/1 mM) and in the absence or presence of α-ketoglutarate (α-KG), glutamate, or malate (all 5 mM). (**C**,**D**) OCR under ATP-supported conditions (10 mM) in RHM (**C**) and permeabilized AC16 cells (**D**) with identical substrate supplementation. CI-linked substrates increased respiratory capacity compared to ATP alone. (**E**,**F**) Quantification of ATP-dependent inhibition at indicated Cyt c concentrations in RHM (**E**) and permeabilized AC16 cells (**F**). ATP inhibition was calculated relative to ADP-supported respiration. In RHM, ATP-dependent inhibition was largely preserved among substrates, whereas in permeabilized AC16 cells, α-KG significantly attenuated ATP-mediated control compared to no-substrate conditions. Data are shown as the mean ± SD (*n* = 3 independent replicates per condition). Statistical analysis was performed using two-way ANOVA with Sidak’s multiple comparisons test. ** *p* < 0.01 and * *p* < 0.05; ns, not significant.

We next examined whether ATP-dependent inhibition of CytOx in RHM and permeabilized AC16 cardiomyocytes is influenced by respiration supported by NADH-linked substrates such as α-ketoglutarate, glutamate and malate. In RHM (left panels), supplementation with α-ketoglutarate, glutamate, or malate increased OCR under ADP conditions (Figure 3A). However, the calculated ATP-dependent inhibition relative to ADP was largely preserved, indicating that CI-linked substrates did not reduce the extent of ATP-mediated control in isolated mitochondria (Figure 3E).

In contrast to isolated mitochondria, permeabilized AC16 cardiomyocytes (right panels) displayed a substrate- and cytochrome c-dependent reduction in ATP-mediated control. In the cardiac-like cells, all three CI-linked substrates significantly reduced ATP-dependent inhibition of CytOx (Figure 3B,F). However, the extent and cytochrome c dependence of this effect differed between substrates. Glutamate attenuated ATP-dependent inhibition already when cytochrome c was not present, whereas α-ketoglutarate and malate reduced ATP inhibition predominantly at higher cytochrome c levels (40 µM). These findings show that substrate-dependent modulation of ATP-mediated control in the cellular context depends on both the metabolic entry point and electron supply.

### 3.3. CI-Linked Substrates α-Ketoglutarate, Glutamate and Malate Differentially Modulate Membrane Potential and ROS Formation

**Figure 4:**
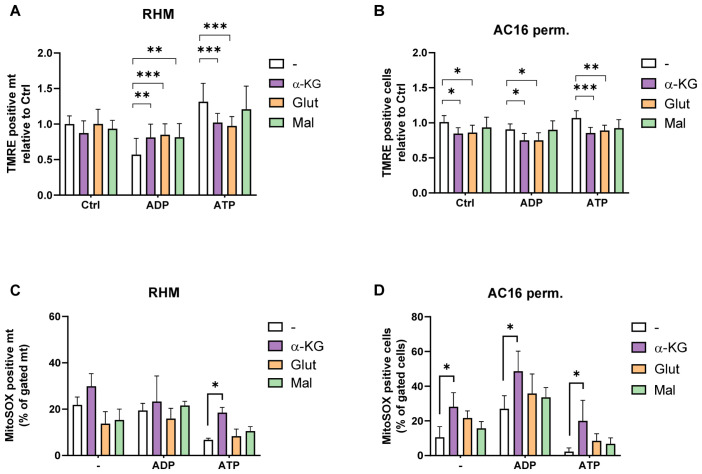
Distinct redox effects of α-ketoglutarate during ATP-dependent respiratory control. (**A**,**B**) Mitochondrial membrane potential (ΔΨm) assessed by TMRE fluorescence in RHM (**A**) and permeabilized AC16 cardiomyocytes (**B**) under control (Ctrl), ADP (1 mM), or ATP (10 mM) conditions in the presence of α-ketoglutarate (α-KG), glutamate (Glu), or malate (Mal); all 5 mM. (**C**,**D**) Mitochondrial superoxide formation quantified by MitoSOX fluorescence in RHM (**C**) and permeabilized AC16 cells (**D**) under identical nucleotide and substrate conditions. For MitoSOX measurements in isolated mitochondria, 0.5 mM malate was included to sustain electron flow. Data are presented as the mean ± SD, (*n* = 3). Statistical analysis was performed using two-way ANOVA and Sidak’s multiple comparisons test. *** *p* < 0.001, ** *p* < 0.01, and * *p* < 0.05.

Given these substrate-dependent differences in ATP-mediated respiratory control, we assessed how these changes are reflected by mitochondrial membrane potential (ΔΨm) and ROS formation (Figure 4).

In RHM, supplementation with CI-linked substrates resulted in modest, but substrate-specific effects (Figure 4A). While α-ketoglutarate and malate slightly attenuated ATP-induced hyperpolarisation, glutamate showed only minor changes. Overall, the ATP-associated increase in ΔΨm remained largely preserved in isolated mitochondria.

In permeabilized AC16 cardiomyocytes, substrate-dependent differences were more pronounced (Figure 4B). In the presence of 10 mM ATP, α-ketoglutarate significantly reduced membrane hyperpolarisation, whereas glutamate and malate had smaller effects. These findings indicate that CI-linked substrates modulate ATP-associated changes in membrane potential stronger in the cellular context than in isolated mitochondria.

We next assessed mitochondrial superoxide formation using MitoSOX (Figure 4C,D). In RHM, CI-linked substrates exerted substrate-specific effects on ROS formation, with α-ketoglutarate significantly increasing mitochondrial superoxide levels under ATP conditions (Figure 4C).

In permeabilized AC16 cardiomyocytes, substrate effects on ROS formation were more pronounced (Figure 4D). α-Ketoglutarate significantly increased mitochondrial superoxide levels under both ADP and ATP conditions compared to control, whereas glutamate and malate showed smaller or intermediate effects. Despite these substrate-dependent differences, ATP was consistently associated with lower ROS production relative to ADP across all substrate conditions.

### 3.4. ATP-Dependent Inhibition Is Preserved in the Presence of Pyruvate, Citrate, Glutamine and Fumarate

**Figure 5:**
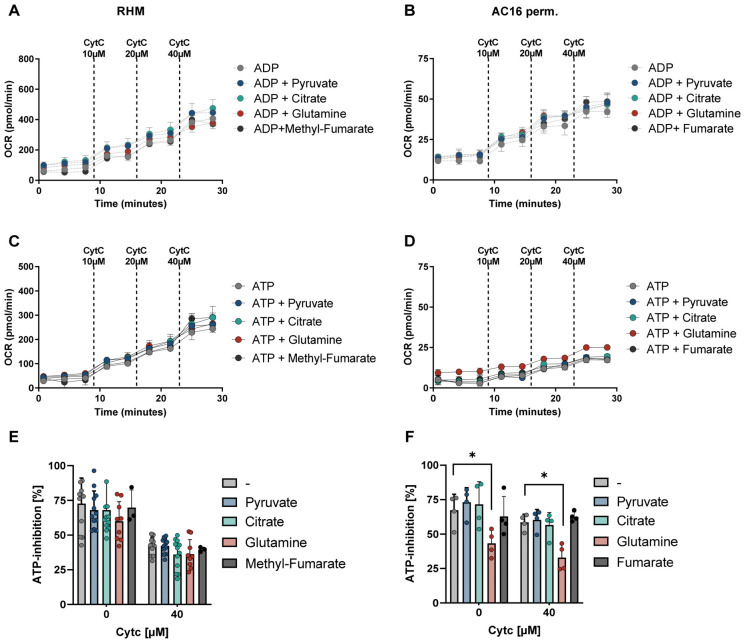
ATP-dependent respiratory control is preserved under moderate CI-linked substrate supply. (**A**,**B**) Representative oxygen consumption rates from isolated RHM (**A**) and permeabilized AC16 cardiomyocytes (**B**) under ADP-supplementation (1 mM). Measurements were performed in the presence of pyruvate, citrate, glutamine, or methyl-fumarate (all 5 mM). (**C**,**D**) Corresponding assessment of respiration profiles under the addition of 10 mM ATP. (**E**,**F**) Quantitative analysis of ATP-dependent inhibition at indicated cytochrome c concentrations in RHM (**E**) and permeabilized AC16 cells (**F**). Data are presented as the mean ± SD. Statistical analysis was performed using two-way ANOVA followed by Sidak’s multiple comparisons test, * *p* < 0.05.

To assess whether the substrate-dependent stimulation of respiration observed with α-ketoglutarate extends to other TCA-related metabolites, oxygen consumption was measured in the presence of pyruvate, citrate, glutamine, or (methyl)-fumarate (Figure 5).

In contrast to α-ketoglutarate, however, ADP supplementation with pyruvate, citrate, glutamine, or methyl-fumarate showed no changes in respiratory rates compared to the condition without substrate (Figure 5A,B). While slight variations were detectable between substrates, none induced a pronounced stimulation of electron flux under these experimental conditions consistent in both model systems.

A similar pattern was observed under ATP-supported respiration. The presence of these metabolites did not markedly shift respiratory capacity relative to ATP alone (Figure 5C,D). Thus, unlike the strong NADH-promoting substrates α-KG or glutamate, these metabolites revealed limited influence on absolute respiratory throughput.

Consistent with these observations, ATP-dependent inhibition of respiration in isolated mitochondria remained largely preserved across substrates (Figure 5E). Although inhibition tended to decline at higher cytochrome c concentrations, this reduction occurred similarly in all groups and was not specifically associated with any individual metabolite.

In permeabilized AC16 cardiomyocytes, substrate-dependent differences were also not apparent (Figure 5F), although glutamine showed a reduction in ATP inhibition, which were not apparent in isolated mitochondria.

Together, these findings indicate that moderate CI-linked electron supply preserves ATP-dependent inhibition in isolated mitochondria but reveals substrate-dependent differences in permeabilized cells.

### 3.5. ATP-Associated Changes in Membrane Potential and ROS Remain Unchanged in the Presence of CI-Linked Substrates Pyruvate, Citrate, Glutamine and Fumarate

**Figure 6:**
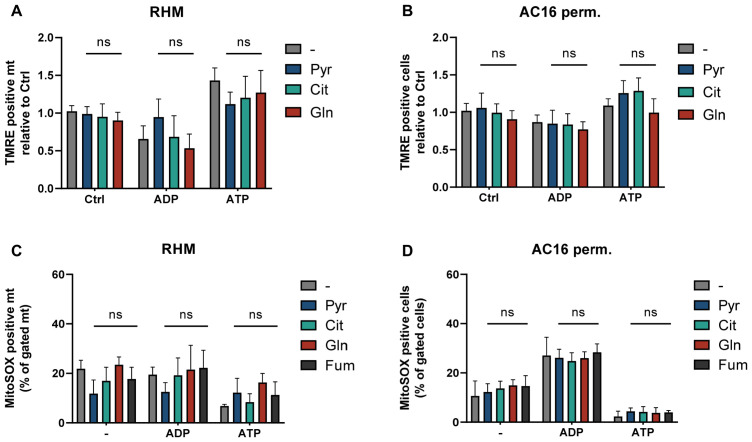
Membrane potential and superoxide levels remain stable across CI-linked substrates pyruvate, citrate, glutamine and fumarate. (**A**,**B**) Mitochondrial membrane potential (ΔΨm) assessed by TMRE fluorescence in RHM (**A**) and permeabilized AC16 cardiomyocytes (**B**) under control (Ctrl), ADP (1 mM), or ATP (10 mM) conditions in the presence of pyruvate (Pyr), citrate (Cit), glutamine (Gln) or fumarate (Fum). Data are expressed relative to control conditions. (**C**,**D**) Mitochondrial superoxide formation quantified by MitoSOX fluorescence in RHM (**C**) and permeabilized AC16 cells (**D**) under identical nucleotide conditions in the presence of pyruvate (Pyr), citrate (Cit), glutamine (Gln), or fumarate (Fum). Data are expressed as percentage of gated mitochondria (RHM) or cells (AC16) and presented as the mean ± SD of three independent replicates per condition (*n* = 3). Statistical analysis was performed using two-way ANOVA followed by Sidak’s multiple comparisons test. ns, not significant.

To determine whether the lack of respiratory stimulation by pyruvate, citrate, glutamine and fumarate (Figure 5) is reflected in mitochondrial functional state, membrane potential was again assessed by TMRE fluorescence in isolated rat heart mitochondria and permeabilized AC16 cardiomyocytes (Figure 6A,B).

In isolated rat heart mitochondria (Figure 6A) none of the tested substrates significantly altered ΔΨm compared to the respective ADP or ATP control conditions. Similarly, in permeabilized AC16 cardiomyocytes (Figure 6B), ATP-induced changes in membrane potential remained also unaffected by substrate supplementation.

Analysis of mitochondrial superoxide production under any nucleotide condition revealed that ROS levels were again not significantly modified in the presence of pyruvate, citrate, glutamine, or fumarate (Figure 6C,D). These findings indicate that, in contrast to α-ketoglutarate, specific CI-linked substrates do not strongly affect ATP-dependent redox regulation.

### 3.6. Succinate-Driven Respiration Bypasses ATP-Dependent Inhibition of Cytochrome c Oxidase

**Figure 7:**
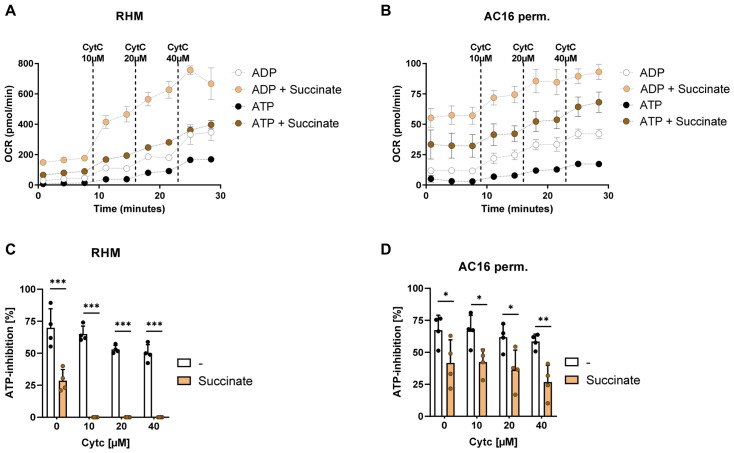
Succinate supplementation attenuates ATP-dependent inhibition of CytOx. (**A**,**B**) Oxygen consumption rates in RHM and permeabilized AC16 cells during stepwise Cyt c titration (10–40 µM) under ADP (1 mM) or ATP (10 mM) conditions in the absence or presence of succinate (5 mM). (**C**,**D**) Quantification of ATP-dependent inhibition in isolated RHM and permeabilized AC16 cells at increasing cytochrome c concentrations in the absence or presence of succinate. ATP inhibition was calculated relative to ADP-dependent respiration. Data are presented as the mean ± SD (n = 4 independent replicates per condition). Statistical significance was determined by two-way ANOVA followed by Sidak’s multiple comparisons test. *** *p* < 0.001, ** *p* < 0.01, and * *p* < 0.05.

To determine whether substrates that feed electrons directly into complex II, influence ATP-dependent respiratory control, oxygen consumption was measured during cytochrome c titration in the presence of succinate (Figure 7).

In isolated rat heart mitochondria, succinate strongly increased respiratory rates under both ADP- and ATP-driven conditions. Respiration increased progressively with rising cytochrome c concentrations, and was particularly pronounced at higher cytochrome c levels, indicating a strong enhancement of mitochondrial electron flux.

A similar pattern was observed in permeabilized AC16 cardiomyocytes. Succinate elevated oxygen consumption among all cytochrome c concentrations under both ADP- and ATP-supported conditions, demonstrating that the effect is not restricted to isolated mitochondria.

To quantify the effect of succinate on regulatory control, ATP inhibition was calculated relative to the corresponding ADP condition.

In isolated mitochondria, ATP inhibition was strong in the absence of substrates, but was significantly reduced in the presence of succinate and increasing cytochrome c levels. While inhibition decreased progressively with increasing cytochrome c even under control conditions, the presence of succinate produced a much stronger reduction, indicating that complex II-supported electron entry diminishes the effectiveness of ATP-dependent control.

Permeabilized AC16 cells showed a similar response. ATP inhibition remained high without substrate, but was consistently lower when succinate was present, confirming that the effect of succinate on ATP regulation is preserved in a cellular context.

### 3.7. CII-Substrate Succinate Differentially Alters ATP-Associated Redox Parameters in a Cell-Dependent Manner

**Figure 8:**
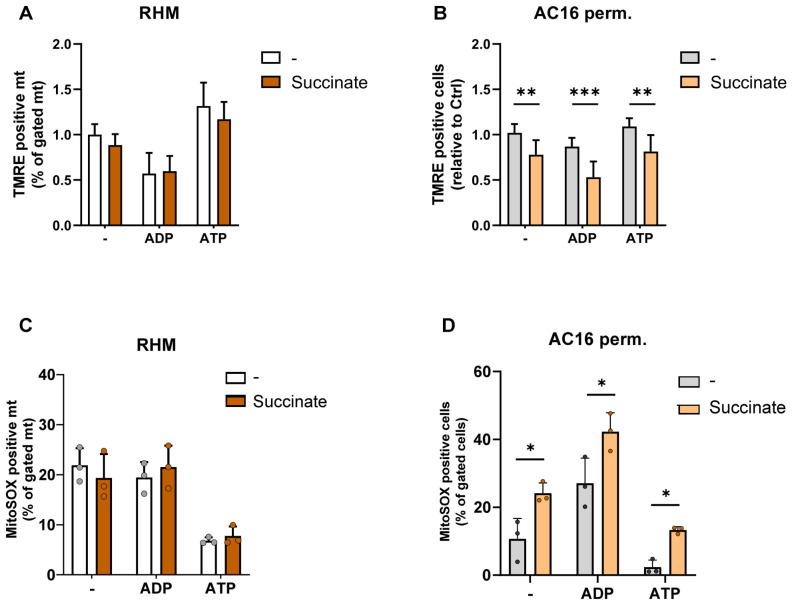
CII-dependent modulation of mitochondrial integrity in isolated rat heart mitochondria and permeabilized AC16 cardiomyocytes. (**A**,**B**) Mitochondrial membrane potential (ΔΨm) and (**C**,**D**) mitochondrial superoxide formation in isolated RHM and permeabilized AC16 cells assessed by TMRE and MitoSOX fluorescence respectively, under control conditions (Ctrl), ADP (1 mM), or ATP (10 mM) in the absence or presence of succinate (10 mM). Data are expressed as percentage of TMRE- or MitoSOX-positive mitochondria/cells. Data are presented as the means ± SD of three replicates per condition. Statistical analysis was performed using two-way ANOVA followed by Sidak’s multiple comparisons test. *** *p* < 0.001, ** *p* < 0.01, and * *p* < 0.05.

To further define the impact of CII-linked electron entry, we assessed ATP-associated changes in membrane potential and ROS formation in the presence of succinate (Figure 8). In isolated rat heart mitochondria, succinate did not significantly affect mitochondrial membrane potential or superoxide formation under control, ADP, or ATP conditions (Figure 8A,C). ATP-induced membrane hyperpolarisation and ATP-associated reduction in ROS were preserved in the presence of succinate.

In contrast, in permeabilized AC16 cardiomyocytes, succinate significantly reduced membrane potential under all nucleotide conditions (Figure 8B). Concomitantly, succinate markedly increased mitochondrial superoxide levels under control, ADP, and ATP conditions (Figure 8D).

These findings indicate that CII-linked electron supply exerts a more pronounced and qualitatively distinct influence on ATP-associated mitochondrial regulation compared to CI-linked substrates.

## 4. Discussion

The present study demonstrates that the inhibition of CytOx by ATP is not a fixed enzymatic process, but rather a regulatory mechanism that is influenced by the rate and mode of electron delivery to the respiratory chain. While ATP consistently reduced oxygen consumption and ROS formation under baseline conditions, specific substrate-driven changes in electron supply increased absolute respiratory activity in isolated mitochondria. In permeabilized cardiomyocytes, they also reduced the magnitude of ATP-dependent inhibition. These findings indicate that the apparent strength of ATP-mediated control results from the interaction between downstream enzymatic regulation at CIV and upstream electron pressure within CI and CII.

### 4.1. ATP-Dependent CIV Inhibition Is Maintained During CI-Linked Respiration in Isolated Mitochondria, but Becomes Substrate-Dependent in Permeabilized Cardiomyocytes

Allosteric ATP binding to CytOx has been proposed to limit electron flux at high ATP/ADP ratios, thereby stabilizing mitochondrial membrane potential and preventing excessive ROS formation [3,9]. Our data confirm the suppression of respiration and reduced ROS release by ATP in two distinct models.

In isolated rat heart mitochondria, CI-linked substrates, including α-ketoglutarate and glutamate, increased absolute respiratory rates under both ADP- and ATP-conditions. Importantly, despite the increase in absolute respiration, the proportional extent of ATP-dependent inhibition remained largely preserved under most CI-linked conditions.

This differential effect may reflect how TCA-related metabolites influence upstream reducing equivalent supply and subsequent electron supply to the respiratory chain. Among the substrates tested, α-ketoglutarate is a particularly potent stimulator of mitochondrial respiration, as its conversion to succinyl-CoA by the α-ketoglutarate dehydrogenase complex (KGDH) generates NADH directly, which feeds electrons into CI [18,19,20]. The KGDH complex represents a major regulatory step within the TCA cycle, exhibiting high catalytic capacity under substrate-rich conditions and enabling rapid NADH production when α-ketoglutarate availability increases [21].

In contrast, the activity of other TCA enzymes, such as malate dehydrogenase and citrate synthase, is more tightly regulated and restricted by substrate equilibrium and feedback inhibition, which limits abrupt NADH accumulation [21,22,23]. Consistent with their differential effects on NADH generation, ATP-dependent inhibition of CytOx remained effective in the presence of pyruvate, citrate or fumarate. However, α-ketoglutarate-driven respiration partially attenuated ATP-mediated control. These findings suggest that the inhibition of CIV by ATP is only effective within a limited range of electron flux. Beyond this range, an increased supply of electrons upstream diminishes its relative impact.

Thus, enhanced NADH generation shifts respiration to a higher absolute flux level without altering the proportional inhibitory effect of ATP on CIV. Consistent with in vivo analyses demonstrating that CytOx exerts significant control over respiratory flux in intact cells [24,25], our data indicate that the regulation of CIV by ATP remains intact even under conditions of elevated NADH-supported flux in isolated mitochondria.

In contrast to isolated mitochondria, permeabilized AC16 cardiomyocytes exhibited a substrate- and cytochrome c-dependent attenuation of ATP-mediated control. Glutamate had an effect on ATP inhibition at low cytochrome c concentrations, whereas α-ketoglutarate and malate predominantly had an effect at higher cytochrome c levels. This suggests that, in the cellular context, differences in upstream electron supply dependent on substrates can influence how strongly ATP-mediated control at CIV becomes functionally apparent.

Moreover, the divergence between isolated mitochondria and permeabilized cells suggests that ATP-dependent CIV inhibition is not solely governed by the intrinsic enzymatic properties but may also depend on the cellular metabolic context. In permeabilized cells, the preservation of structural and metabolic organisation may alter substrate accessibility and local metabolic coupling, thereby, modulating the distribution of respiratory flux control [26,27,28]. Such effects may involve more efficient transfer of TCA-related intermediates and reducing equivalents between matrix dehydrogenases and the respiratory chain. This transfer may be better preserved in permeabilized cells than in isolated mitochondria, which could explain why ATP-mediated control at CytOx appears to be more flexible in permeabilized cardiomyocytes.

### 4.2. CII-Linked Respiration Attenuates ATP-Dependent CIV Inhibition

Unlike CI-linked substrates, electron entry via complex II altered the proportional impact of ATP on respiration. Succinate-supported respiration significantly reduced the relative magnitude of ATP-dependent inhibition in both isolated mitochondria and permeabilized cardiomyocytes. Although ATP continued to suppress oxygen consumption, the proportional reduction was markedly attenuated compared to conditions without succinate. Importantly, this attenuation was observed at every cytochrome c concentration, indicating that the electron supply driven by CII diminishes the functional effectiveness of ATP-mediated regulation at CIV.

In contrast to CI-linked respiration, which generates NADH and drives proton pumping and membrane potential formation, CII feeds electrons directly into the ubiquinone pool without translocating protons. This establishes a distinct redox configuration within the respiratory chain [29,30]. Under these conditions, ATP-dependent inhibition of CIV appears to become proportionally less dominant, suggesting that the site of electron entry influences the relative distribution of control within the electron transport chain.

These findings are consistent with the principles of metabolic control analysis, which state that respiratory flux control is distributed among multiple enzymatic steps and depends on the metabolic context [31,32,33]. In intact human cells, CytOx has been shown to exert pronounced control over respiratory flux, particularly when its activity becomes rate-limiting [24,25]. Our data build on this concept by suggesting that the control coefficient of CIV varies depending on the mode and intensity of electron supply, especially under CI- versus CII-supported respiration.

### 4.3. α-Ketoglutarate Links NADH Flux to ROS Generation Independent of ΔΨm

Among the substrates linked to CI, α-ketoglutarate produced the strongest increase in mitochondrial ROS formation. This finding is consistent with previous work showing that α-ketoglutarate dehydrogenase (KGDH) is not only a major NADH-generating enzyme of the TCA cycle, but can also act as a mitochondrial ROS production site, particularly under highly reduced conditions [19,34,35]. Although the precise site of ROS generation and NADH/NAD^+^ ratios were not directly assessed in the present study, enhanced α-ketoglutarate metabolism may have increased upstream reducing pressure, thereby contributing to the elevated ROS formation observed under these conditions [27].

Notably, the increase in ROS observed under α-ketoglutarate was not accompanied by changes in membrane potential. This suggests that ROS production under these conditions is predominantly driven by the redox state of electron carriers rather than hyperpolarisation alone. Although elevated ΔΨm can enhance electron leakage at complexes I and III [15,36], experimental and mechanistic analyses indicate that high NADH/NAD^+^ ratios and increased reduction of the Q-pool can independently promote superoxide formation, even in the absence of significant membrane hyperpolarisation [27,37]. Thus, under CI-linked respiration, ATP-dependent inhibition of CIV does not necessarily prevent ROS formation when NADH generation is high upstream, highlighting that redox pressure rather than ΔΨm may represent the dominant determinant of superoxide production in this context.

In contrast to α-ketoglutarate and glutamate, substrates such as pyruvate, citrate, glutamine, and fumarate did not notably increase ROS formation and preserved proportional ATP-dependent respiratory suppression. These variations may reflect differences in the capacity of the tested substrates to support the supply of reducing equivalents upstream, as well as differences in metabolic coupling between isolated mitochondria and permeabilized cells depending on the system. Enzymes such as malate dehydrogenase and citrate synthase are more tightly regulated by substrate equilibrium and feedback mechanisms, thereby limiting abrupt NADH accumulation and excessive reduction pressure [18,21]. Consequently, the impact of ATP-dependent CIV inhibition on ROS formation reflects the existing upstream redox state rather than altered ATP sensitivity.

### 4.4. CII-Linked Respiration Uncouples ROS Formation from Membrane Potential Changes

In contrast to CI-driven ROS formation, CII-linked respiration revealed a distinct redox pattern. This demonstrates that the site of electron entry influences both respiratory flux control and the mechanistic origin of superoxide generation [30,38].

In isolated rat heart mitochondria, succinate supplementation did not significantly alter the membrane potential or mitochondrial ROS formation under ADP- or ATP-driven conditions. Despite attenuating ATP-dependent respiratory inhibition, CII-linked electron supply did not promote superoxide accumulation in this system, indicating that proportional control of the redox balance remained largely preserved. Such context-dependent effects of succinate on ROS production have been reported previously and depend on the predominant redox state and proton motive force [17,27].

In contrast, permeabilized AC16 cardiomyocytes exhibited a distinct response. Succinate significantly increased mitochondrial superoxide levels under both ADP- and ATP-dependent conditions, despite a concomitant reduction in membrane potential. This dissociation between ΔΨm and ROS formation suggests that, during CII-linked respiration, superoxide generation may not be driven solely by hyperpolarisation and may instead reflect changes in the ubiquinone pool and the distribution of electrons within the respiratory chain [16,39,40]. Increased reduction of the Q-pool has been shown to enhance electron leak at specific sites of the respiratory chain, independently of the magnitude of ΔΨm [27,38].

As CII feeds electrons directly into the ubiquinone pool without contributing to proton pumping, the redox configuration of succinate-supported respiration differs from that of NADH-driven flux [29,30]. Under conditions of substantial Q-pool reduction combined with a high membrane potential, reverse electron transport (RET) can occur from CII to CI, resulting in pronounced superoxide production at CI [16,17]. However, in the present study, succinate supplementation in permeabilized cardiomyocytes was associated with a reduction rather than an increase in ΔΨm. This observation makes classical RET-driven ROS generation less likely to represent the dominant mechanism under our experimental conditions and instead points towards forward electron leak mechanisms driven by altered redox distribution within the chain.

### 4.5. System-Dependent Differences

The more pronounced substrate effects observed in permeabilised cardiomyocytes than in isolated mitochondria likely reflect the preservation of metabolic coupling, compartmentalised redox pools, and interactions between the cytosol and mitochondria within the cellular context. In intact or semi-intact systems, the mitochondrial NADH/NAD^+^ balance is influenced by matrix dehydrogenase activity, substrate availability, and the transfer of cytosolic reducing equivalents via redox shuttles, such as the malate-aspartate shuttle. Together, these factors determine the overall redox pressure exerted on the electron transport chain [28,41,42,43].

In contrast, although isolated mitochondria are experimentally tractable, they lack aspects of intracellular structural organization and metabolic integration. Disruption of cellular structure during isolation may alter the accessibility of TCA-related substrates and the local mitochondrial redox environment. Additionally, loss of cellular organization may reduce metabolic coupling between mitochondria and surrounding compartments, contributing to the observed differences between isolated mitochondria and permeabilized cells. All of these factors have been shown to influence the respiratory flux and the relative contribution of individual complexes to overall control [28,44].

Accordingly, the greater variability observed in the inhibition of CIV by ATP in permeabilized cardiomyocytes likely reflects a more integrated metabolic environment, in which electron supply, redox state and respiratory control are more closely coupled. These system-dependent differences emphasise that the functional expression of ATP-mediated regulation cannot be fully understood in isolation from the structural and metabolic context in which the respiratory chain operates.

In this context, it has been demonstrated that respiratory control is further driven by the transfer of protons linked to the formation of metabolic water at CytOx [2,27]. This proton transfer is mediated by the complexes I, III, and IV, which couple electron flow to proton translocation across the inner mitochondrial membrane in order to generate the proton motive force. Importantly, oxygen reduction directly produces matrix water, thereby linking respiratory activity to local hydrogen availability and to inter- and intramolecular differences in hydrogen isotope distribution. Accordingly, the hydrogen isotopic composition of metabolic substrates may influence proton-coupled reactions within the respiratory chain. Different nutrient classes exhibit distinct ^2^H/^1^H ratios, which may result in variable hydrogen isotope enrichment during substrate oxidation [45,46]. Although deuterium is present at relatively low natural abundance, its physiological concentration is not negligible and may affect proton transfer kinetics due to isotope-dependent differences in dissociation and mobility [47,48]. Such effects may influence proton-binding residues within the ATP synthase proton channel [49], while water-mediated hydrogen exchange reactions may further modulate proton availability at this site. This could potentially affect the efficiency of proton translocation and ATP synthesis. From this perspective, substrate-dependent differences in hydrogen composition and metabolic water production could restrict proton transfer processes and influence mitochondrial function.

When applied to the present study, these concepts potentially explain the observed discrepancies between isolated mitochondria and permeabilized cardiomyocytes. In permeabilized cells, preserving glycolysis, carbon metabolism, and the tricarboxylic acid cycle may facilitate continuous substrate turnover, metabolic water exchange, and deuterium redistribution. This may contribute to compartmental and molecular differences in hydrogen isotope composition and more dynamic proton transfer conditions [50,51,52]. By contrast, these processes are largely absent in isolated mitochondria, leading to more uniform proton transfer dynamics and contributing to the observed variations in the inhibition of cytochrome c oxidase by ATP.

However, these mechanisms were not directly addressed in the present study. Although it has been proposed that substrate-dependent hydrogen composition and metabolic water formation restrict proton transfer dynamics under physiological conditions, our experimental approach did not include assessing proton transfer pathways, metabolic water composition or isotopic effects. Consequently, the contribution of such processes cannot be resolved within our system.

### 4.6. Physiological Implications for Cardiac Metabolism

Cardiac metabolism is characterized by rapid shifts in substrate utilization and redox state in response to workload and oxygen availability. During ischemia–reperfusion or metabolic remodeling, the accumulation of NADH and succinate can temporarily elevate electron pressure within the electron transport chain [16,53]. The rapid oxidation of these reducing equivalents upon reperfusion has been linked to enhanced ROS formation and altered redox distribution [15,16].

Our findings suggest that under such conditions, the effectiveness of ATP-dependent inhibition of CytOx depends on the predominant mode of electron entry. An increased supply of NADH via CI-linked substrates can raise redox pressure while maintaining proportional CIV control within a defined flux range. In contrast, enhanced succinate oxidation via CII may redistribute respiratory control and reduce the contribution of CIV-mediated regulation to ROS dynamics.

Complex II plays a central role in shaping cardiac mitochondrial redox balance. During ischemia–reperfusion, succinate accumulation and its rapid oxidation upon reperfusion drive enhanced electron flux through the ubiquinone pool and promote ROS formation via reverse electron transport. These effects can be mitigated by pharmacological inhibition of CII with malonate [54,55]. Furthermore, CII activity is a major component of the reserve respiratory capacity required to sustain ATP production under stressful conditions [56]. Our data further suggest that increased electron supply via CII can override ATP-dependent CIV inhibition, shifting respiratory control upstream within the electron transport chain. This indicates that metabolic states characterized by elevated succinate oxidation may weaken the effectiveness of ATP-mediated feedback at CIV, thereby promoting increased electron flux through the respiratory chain and potentially increasing redox pressure within it.

## 5. Limitations

In our study, we examined the acute, substrate-dependent effects under defined ATP/ADP ratios. We did not directly quantify the redox state of the ubiquinone pool, the structural organization of respiratory supercomplexes, or potential post-translational modifications of CIV. These parameters may further influence the extent of ATP-dependent respiratory control in vivo. Furthermore, we used two experimental systems: isolated rat heart mitochondria and permeabilized AC16 cardiomyocytes, which differ in structural integrity and metabolic context. Isolation procedures may affect mitochondrial structure and have been reported to alter CI stability and supercomplex organization [42,47,48,49]. Although permeabilized cells preserve aspects of intracellular organization and protein interactions, they still represent a controlled environment in which substrates and adenine nucleotides are imposed experimentally [50,51]. Thus, in both systems, mitochondrial respiration was assessed under defined, non-physiological substrate conditions.

In addition, electron flux was not matched across different substrate conditions in our experiments. While normalising electron transfer rates would allow for a more direct comparison of ATP-dependent regulation at constant flux, implementing this approach across substrates that enter the respiratory chain at different sites is challenging. Moreover, differences in electron supply dependent on the substrate may themselves represent a physiologically relevant regulatory dimension rather than a confounding factor. Therefore, our findings suggest that the effectiveness of ATP-dependent CytOx inhibition is determined by both the magnitude of electron flux and the entry point of reducing equivalents into the respiratory chain.

Despite these challenges, key observations were consistently made across both model systems, particularly with regard to the attenuation of ATP-dependent CIV inhibition under CII-linked respiration. These observations support the robustness of the substrate-dependent effects described here.

## 6. Conclusions

Overall, our findings reveal a mechanistic link between the allosteric inhibition of cytochrome c oxidase by ATP and the metabolism of the TCA cycle, providing insight into how specific metabolites influence mitochondrial respiratory control. In cardiac mitochondria, where substrate availability adapts dynamically to workload, pathological shifts in substrate utilization may alter the effectiveness of ATP-dependent CIV inhibition. When sustained, such shifts could weaken this intrinsic feedback mechanism and thereby contribute to oxidative stress and dysregulated electron transport chain activity.

## Data Availability

The original contributions presented in this study are included in the article. Further inquiries can be directed to the corresponding author.
